# VEGF Signaling Pathway Germline Polymorphisms as Prognostic Pharmacogenetic Biomarkers in Localized High-Grade Osteosarcoma Patients from the GEIS-33 Protocol

**DOI:** 10.3390/ph18121855

**Published:** 2025-12-05

**Authors:** Juliana Salazar, María J. Arranz, Javier Martin-Broto, Massimo Serra, Emanuela Palmerini, Jeronimo Martínez-García, Antonio Juan-Ribelles, Maitane Andion, Javier Martinez-Trufero, Guiomar Gutiérrez, Aizpea Echebarria-Barona, Maria A. Vaz-Salgado, Alexandra Regueiro, Rosa Alvarez, Claudia Valverde, Pablo Luna, Pilar Blay, Ana Sebio

**Affiliations:** 1 Translational Medical Oncology Laboratory, Institut de Recerca Sant Pau (IR Sant Pau), 08041 Barcelona, Spain; 2 Research Laboratory Unit, Fundació Docència i Recerca Mútua Terrassa, 08221 Terrassa, Spain; 3 Medical Oncology Department, Hospital Universitario Fundación Jiménez Díaz, 28040 Madrid, Spain; 4 Osteoncology, Bone and Soft Tissue Sarcomas and Innovative Therapies Unit, IRCCS Istituto Ortopedico Rizzoli, 40136 Bologna, Italy; massimo.serra@ior.it (M.S.);; 5 Sylvester Comprehensive Cancer Center, University of Miami Miller School of Medicine, Miami, FL 33136, USA; 6 Medical Oncology Department, Hospital Universitario Virgen de la Arrixaca, 30120 El Palmar, Spain; 7 Pediatric Oncology and Hematology Unit, Hospital Universitario y Politécnico La Fe de Valencia, 46026 Valencia, Spain; 8 Pediatric Hematology and Oncology Department, Hospital Niño Jesús, 28009 Madrid, Spain; maitane.andion@salud.madrid.org; 9 Medical Oncology Department, University Hospital Miguel Servet, 50009 Zaragoza, Spain; 10 Hospital Regional Universitario de Málaga, 29010 Málaga, Spain; 11 Pediatric Oncology Group, Pediatrics Department, Hospital Universitario Cruces, 48940 Barakaldo, Spain; 12 Medical Oncology Department, Hospital Universitario Ramón y Cajal, 28034 Madrid, Spain; 13 Medical Oncology Department, Complejo Hospitalario Universitario de Santiago de Compostela, 15706 Santiago de Compostela, Spain; 14 Medical Oncology Department, Hospital Universitario Gregorio Marañón, 28007 Madrid, Spain; rosa.alvarez.al@gmail.com; 15 Medical Oncology Department, Vall d’Hebrón Institute of Oncology (VHIO), Hospital Universitari Vall d’Hebrón, 08035 Barcelona, Spain; 16 Medical Oncology Department, Hospital Universitari Son Espases, 07120 Palma, Spain; 17 Medical Oncology Department, Hospital Universitario Central de Asturias, 33011 Oviedo, Spain; 18 Medical Oncology Department, Hospital de la Santa Creu i Sant Pau, 08041 Barcelona, Spain

**Keywords:** pharmacogenetics, osteosarcoma, VEGF, polymorphism

## Abstract

**Background/Objective:** The vascular endothelial growth factor (VEGF) signaling pathway induces angiogenesis, which impacts tumor progression and clinical outcomes in patients with localized osteosarcoma. This study evaluates whether genetic polymorphisms in the VEGF signaling pathway are associated with survival outcomes in these patients. **Methods:** Sixty-nine patients with localized high-grade osteosarcoma enrolled in the GEIS-33 protocol and treated with MAP (methotrexate, doxorubicin, cisplatin) chemotherapy, surgery, and subsequent adjuvant treatment were included. Nine variants of interest in the *VEGFA* (rs1570360, rs2010963 and rs699947), *FLT1* (VEGFR1; rs7993418, rs9513070 and rs9582036), and *KDR* (VEGFR2; rs1551641 and rs1870377 and rs2071559) genes were genotyped from peripheral blood samples using TaqMan Assay technology. Genetic data were correlated with relapse-free survival (RFS) and overall survival (OS) considering clinical variables as covariates. **Results:** The analyses showed nominally significant associations between the *FLT1* variants rs7993418 and rs9582036 and survival. Patients carrying the rs7993418(C) allele had worse RFS (*p* = 0.01) and OS (*p* = 0.01). Carriers of the minor rs9582036(C) allele also had worse RFS (*p* = 0.02) and OS (*p* = 0.03). Additionally, patients harboring the TT genotype of the *KDR* rs1551641 variant had significantly worse RFS (*p* = 0.002). These polymorphisms remained statistically significant in the multivariate Cox regression analyses that included surgical margins and pathological response as covariates. **Conclusions:** Pharmacogenetics may contribute to precision medicine in oncology. Germline polymorphisms in the VEGF pathway may be useful as predictors of survival in high-grade localized osteosarcoma patients treated with chemotherapy, following validation in a large cohort of patients. Current treatment strategies aimed at improving outcomes for osteosarcoma patients may benefit from the identification of new biomarkers, such as these *FLT1* rs7993418 and rs9582036 variants.

## 1. Introduction

High-grade osteosarcoma is a rare malignant tumor with aggressive clinical behavior. The 5-year overall survival in patients with localized osteosarcoma remains stable at around 60–70% [1], regardless of a multidisciplinary approach comprising surgery and multidrug chemotherapy regimens [2]. Tumor site and size, primary metastases, pathological response to neoadjuvant chemotherapy and surgical resection margins are major predictors for long-term survival [3]. However, these factors are insufficient to accurately predict survival. Improving survival in osteosarcoma patients relies on the development of new targeted therapies and on the identification of biomarkers for patient stratification and treatment selection.

Neovascularization is fundamental for the progression of solid tumors. The vascular endothelial growth factor (VEGF, also known as VEGF-A) signaling pathway, including its receptors VEGFR1 and VEGFR2, promotes angiogenesis by activating the autophosphorylation of tyrosine residues and a kinase cascade [4]. These angiogenic processes contribute to the development and progression of osteosarcoma [5]. Additionally, VEGF has an immunosuppressive effect on the tumor microenvironment. High levels of VEGF limit the infiltration and function of cytotoxic T lymphocytes, inhibit dendritic cell maturation, and promote the accumulation of immunosuppressive cells such as myeloid-derived suppressor cells and regulatory T cells [6]. Because of the clinical relevance of the alterations in the VEGF signaling, inhibitors of this pathway, such as tyrosine kinase receptors, are currently being investigated in clinical trials [7,8,9] in those patients diagnosed with osteosarcoma who have previously progressed to chemotherapy [10,11] and as maintenance treatment after complete remission [12].

In osteosarcoma patients, tumor overexpression of the VEGF protein has been associated with poor prognosis [13,14], and the amplification of the VEGF or VEGFR2 coding genes has also been reported in these patients [14,15,16]. In addition to somatic copy-number alterations, common genetic variants in VEGF pathway genes have been studied in relation to disease susceptibility [17,18] and prognosis [19,20]. Polymorphisms in the VEGF signaling pathway may influence angiogenic processes and, consequently, affect clinical outcomes in patients with osteosarcoma [21]. Therefore, introducing pharmacogenetic biomarkers alongside traditional clinicopathological features could improve clinical decision-making for these patients. However, studies addressing these genes as potential biomarkers of survival in high-grade osteosarcoma are very limited [19,20].

The aim of this study was to evaluate the prognostic value of germline polymorphisms in key genes of the VEGF signaling pathway (*VEGFA*, *FLT1* and *KDR*) in a prospective multicenter cohort of patients with localized high-grade osteosarcoma treated with the GEIS-33 protocol.

## 2. Results

### 2.1. Clinical Results

Descriptive data of patients and clinical characteristics are summarized in Table S1. Patients were followed for a median of 62.4 months (interquartile range [IQR]: 38.4–80.3). Median recurrence-free survival (RFS) and overall survival (OS) could not be determined at the data cut-off. The 5-year RFS was 71% (95% CI 57–85%), and the 5-year OS was 72% (95% CI 58–85%).

The clinical variants analyzed showed no significant differences in OS: surgical margins (wide/radical versus marginal; *p* = 0.11), pathological response (tumor necrosis ≥ 90% versus tumor necrosis < 90%; *p* = 0.17), age (4–10 years versus 10–32 years; *p* = 0.8), gender (*p* = 0.22) and tumor site (femur/humerus versus other; *p* = 0.7). In contrast, surgical margins were significantly associated with RFS (*p* = 0.04). The 5-year estimated RFS rate for patients undergoing wide or radical surgical margins was 83% (95% CI 71–95%) and for those with marginal surgical margins was 39% (95% CI NA–80%). Surgical margins were then included as a covariate in the multivariate Cox models, along with pathological response, as tumor necrosis is the only parameter directly related to chemotherapy responsiveness.

### 2.2. VEGF Pathway Polymorphisms and Recurrence-Free Survival

Univariate analyses revealed significant nominal associations between RFS and polymorphisms in the *FLT1* (rs7993418 T > C and rs9582036 A > C) and in the *KDR* (rs1551641 C > T) genes (Table 1).

**Figure 1:**
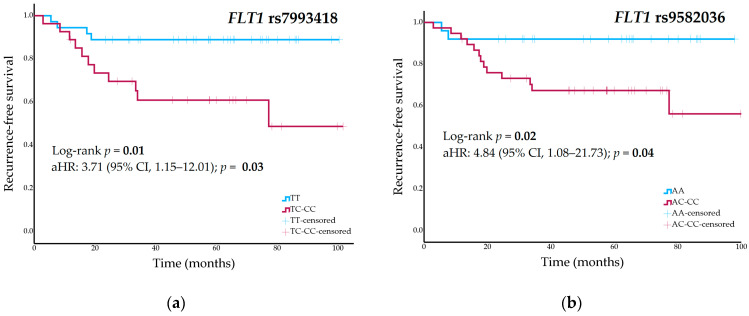
(**a**) Recurrence-free survival curves stratified by *FLT1* rs7993418 genotype (dominant model); (**b**) Recurrence-free survival curves stratified by *FLT1* rs9582036 genotype (dominant model) in the cohort of high-grade osteosarcoma patients.

The 5-year RFS was 89% (95% CI, 79–99%) for *FLT1* rs7993418-TT genotype carriers, 48% (95% CI, 21–75%) for heterozygous patients, and 50% (95% CI, NA–NA%) for patients with CC genotype (*p* = 0.03 in a codominant model and *p* = 0.01 in a dominant model) (Figure 1a). Participants with *FLT1* rs9582036-AA genotype had a 5-year RFS of 92% (95% CI, 82–NA%), compared to a rate of 58% (95% CI, 36–80%) in carriers of the C allele (*p* = 0.02 in a dominant model) (Figure 1b). Carriers of the C allele in *KDR* rs1551641 had a 5-year RFS of 76% (95% CI, 60–92%), while those patients with TT genotype had a rate of 33% (95% CI, NA–70%) (*p* = 0.002 in a recessive model). Table S2 summarizes univariate analysis between all genetic variants and RFS.

Cox regression analyses of significant nominal findings in the univariate analyses including surgical margins and pathological response as covariates showed nominal associations with RFS: *FLT1* rs7993418 [adjusted HR (aHR), 3.71; 95% CI, 1.15–12.01; *p* = 0.03], *FLT1* rs9582036 (aHR, 4.84; 95% CI, 1.08–21.73; *p* = 0.04), and *KDR* rs1551641 (aHR, 7.34; 95% CI, 2.07–26.03; *p* = 0.002).

### 2.3. VEGF Pathway Polymorphisms and Overall Survival

**Figure 2:**
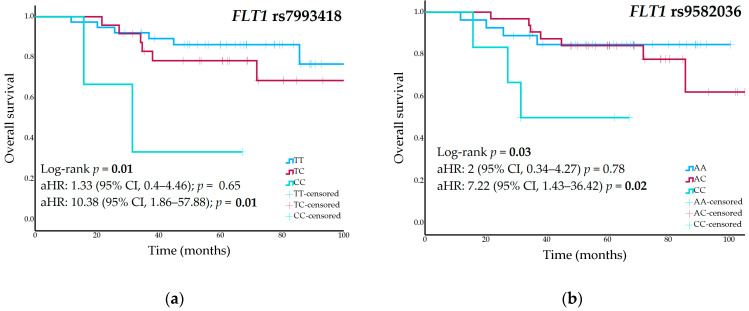
(**a**) Overall survival curves stratified by *FLT1* rs7993418 genotype; (**b**) Overall survival curves stratified by *FLT1* rs9582036 genotype in the cohort of high-grade osteosarcoma patients.

Nominal associations were identified in the univariate analyses between OS and the *FLT1* rs7993418 and rs9582036 variants (Table 1). The 5-year OS was 79% (95% CI, 61–97%) for TT genotype carriers of the rs7993418 variant, 68% (95% CI, 44–92%) for heterozygous patients, and 33% (95% CI, NA–86%) for patients with the CC genotype (*p* = 0.01) (Figure 2a). Patients with the rs9582036-AA genotype had a 5-year RFS of 84% (95% CI, 70–98%), those with the AC genotype had an RFS of 68% (95% CI, 46–90%), and patients with the CC genotype had a rate of 46% (95% CI, 4–87%) (*p* = 0.03) (Figure 2b). Table S3 shows univariate analyses of all genetic variants and OS.

These polymorphisms remained statistically significant in the multivariate Cox regression analyses of OS, adjusted for surgical margins and pathological response. *FLT1* rs7993418 heterozygous patients showed an increased risk of death (aHR, 1.33; 95% CI, 0.4–4.46; *p* = 0.65), while homozygous CC patients had a significantly higher risk (aHR, 10.38; 95% CI, 1.86–57.88; *p* = 0.01). *FLT1* rs9582036 heterozygous patients showed an increased risk of death (aHR, 2; 95% CI, 0.34–4.27; *p* = 0.78), which was significantly higher in homozygous CC patients (aHR, 7.22; 95% CI, 1.43–36.42; *p* = 0.02).

### 2.4. Cumulative Effects of FLT1 rs7993418 and rs9582036 Variants on Survival

Given that two polymorphisms in the same gene, *FLT1* rs7993418 and rs9582036, were found in association with OS and RFS, their cumulative effect was investigated.

*FLT1* rs7993418 and rs9582036 variants were, as expected, in moderately strong linkage disequilibrium (D’= 1.0 and r2 = 0.59). The cumulative effect of the two variants on survival was assessed by stratifying patients into three groups according to their genotypes: wild-type for both variants (group 1; n = 25), wild-type for rs7993418 and minor allele carriers for rs9582036 (group 2; n = 11), and carriers of the minor allele of both variants (group 3; n = 27). No patients were wild-type for rs9582036 and carriers of the minor allele for rs7993418. Kaplan-Meyer analyses showed a statistically significant effect on RFS (*p* = 0.02), but not on OS (*p* = 0.4). When considering the covariates surgical margins and pathological response in the RFS analyses, group 2 (HR, 3; 95% CI, 0.41–21.94; *p* = 0.28) and group 3 (HR, 5.5; 95% CI, 1.2–22.25; *p* = 0.03) had an increased risk of the event.

### 2.5. VEGF Pathway Polymorphisms and Chemotherapy Response

No statistically significant associations with pathological response were observed in the univariate analyses of the *FLT1* rs7993418 and rs9582036 variants, or the *KDR* rs1551641 variant.

## 3. Discussion

The present study investigated the prognostic value of genetic variants in the VEGF signaling pathway in patients with localized high-grade osteosarcoma. Our results provide evidence of the associations between *FLT1* genetic variants rs7993418 and rs9582036, and the *KDR* rs1551641 variant, with survival.

This study is the first one to show significant associations between RFS and OS and two variants in the *FLT1* gene in patients with localized osteosarcoma. Patients homozygous for the minor allele at rs7993418 or rs9582036 variants had lower survival rates. *FLT1* rs9582036 A > C is an intron variant that has been shown to confer a decreased transcriptional activity in endothelial cells [22]. In contrast, *FLT1* rs7993418 T > C is a synonymous substitution in exon 28 that changes the TAT codon to TAC at tyrosine 1213 (Tyr1213Tyr), a residue identified as one of the major VEGFR1 autophosphorylation sites. Lambrechts et al. [23] showed that the TAC codon produced similar mRNA expression levels to that of the wild-type codon, but led to an increase in both soluble (sVEGFR1) and transmembrane forms of VEGFR1 protein levels. As a consequence, they observed an amplification of downstream VEGFR1 signaling in response to activation by placental growth factor (PGF), a VEGF homolog. The contrasting putative functionalities described for these two variants highlight the need for further functional studies, and reflect the complexity of the VEGFR1 role in angiogenesis. sVEGFR1 acts as a decoy receptor for VEGF, downregulating VEGFR2-mediated angiogenesis [24], whereas VEGFR1 activates downstream signaling upon ligand binding, either by VEGF or by PGF [25].

Our *FLT1* rs9582036 finding agrees with the results of previous cancer association studies. Several studies focusing on biomarkers of bevacizumab treatment have reported a poor prognosis for carriers of the minor C allele in metastatic renal-cell carcinoma (mRCC) and pancreatic [23], colorectal (mCRC) [26] or breast [27] cancers. Studies have also associated the C allele with worse survival outcomes in mRCC patients treated with sunitinib [28,29]. This association was also reported in stage I-III non-small cell lung cancer (NSCLC) patients who received surgical treatment [22]. In addition, the minor allele of the *FLT1* rs7993418 variant has been corelated with poor survival in mRCC patients treated with bevacizumab [23] or sunitinib [28]. This association was also observed in advanced or recurrent NSCLC patients treated with bevacizumab and chemotherapy who were carriers of the *FLT1* rs9554316(T) allele [30], which is correlated with the rs7993418(C) allele (D’ = 1.0, r2 = 0.98). However, other studies have not found any association between rs9582036 or rs7993418 variants and survival [31,32,33].

Due to the heterogeneity of cancer types and treatments, including anti-angiogenic therapies, surgery, and chemotherapy, comparing results and drawing conclusions from the above-mentioned studies remains difficult. However, these findings, together with ours, suggest that the rs9582036 and rs7993418 variants in the *FLT1* gene represent promising prognostic factors, although further research in required. These *FLT1* genetic variants may also be investigated as candidate predictive biomarkers for anti-angiogenic therapies in future research. Prospective clinical trials evaluating multi-target tyrosine kinase inhibitors in monotherapy, mainly in patients with relapsed or refractory osteosarcoma, demonstrated modest efficacy, and frequent dose interruptions and reductions due to toxicity [10,11]. Other treatment strategies involve multi-tyrosine kinase inhibitors followed by standard chemotherapy in newly diagnosed high-risk patients [12], or as part of maintenance in the relapsed setting [34]. All these therapeutic approaches could benefit from the evaluation of predictive biomarkers, such as *FLT1* rs9582036 or rs7993418, to better identify patients most likely to respond to antiangiogenic treatments. The discovery of pharmacogenetic biomarkers for chemotherapy and novel targeted cancer therapies could improve clinical outcomes by facilitating more effective treatments, and, ultimately, contribute to the progress of precision oncology.

This study additionally revealed that the minor *KDR* rs1551641(T) allele was associated with worse RFS. There are no functional studies on this variant to date. However, prediction scores from the Regulome database (rank 1f, score 0.22) suggest it may have regulatory activity. Several studies have analyzed this variant, or *KDR* rs10013228, a promoter variant that is in high linkage disequilibrium with the former, as a prognostic factor, but the results are inconsistent. In a study conducted in mCRC patients, homozygous carriers of the *KDR* rs1551641(T) allele showed increased OS [35]. The *KDR* rs10013228(G) allele (linked to the rs1551641(T) allele) has been associated with lower risk of recurrence in patients with CRC [36], but with worse disease-free survival and OS in patients with RCC [37].

Several limitations of our study should be addressed. Although the sample size was not large, it has to be underlined that it was a homogeneous cohort obtained from a multicenter clinical trial, and the statistical power was appropriate to address the study aims. It is worth mentioning that the missing data (4 participants for OS and 6 for RFS), along with the lack of sensitivity analyses, may have introduced potential biases. The hazard ratios were unstable, with wide confidence intervals, probably due to the complex nature of the disease traits. Validation in an independent cohort and further functional studies are required to support the *FLT1* variants rs7993418 and rs9582036 as prognostic biomarkers in osteosarcoma disease. Future research should also include additional structural and regulatory SNPs of genes on the VEGF signaling pathway to better discern their possible effect on the prognosis of these patients. Finally, the significant findings did not survive the Bonferroni correction for multiple comparisons. However, the Bonferroni corrections is a strict method that does not account for the linkage disequilibrium structure of variants within the same gene or the hypothesis-driven selection of the variants.

In conclusion, *FLT1* rs7993418 and rs9582036 variants may be prognostic biomarkers of survival in high-grade localized osteosarcoma treated with chemotherapy. These findings, if confirmed, may support treatment strategies for patients with localized osteosarcoma, as long-term survival rates have plateaued since the introduction of intensive chemotherapy regimens, and the clinicopathological factors that contribute to prognosis do not fully predict individual patient survival at the time of diagnosis.

## 4. Materials and Methods

### 4.1. Study Design and Population

This prospective study included 69 patients diagnosed with localized high-grade osteosarcoma. Patients were included in the GEIS-33 (Spanish Research Sarcoma Group) multicenter protocol between July 2016 and November 2020. Patients received two cycles of high-dose methotrexate 12 g/m^2^, doxorubicin 90 mg/m^2^ (Adriamycin) and cisplatin 120 mg/m^2^ (known as MAP chemotherapy) followed by surgery. Subsequently, adjuvant treatment was tailored based on P-glycoprotein expression at diagnosis and histological response.

P-glycoprotein determination was conducted at the Istituto Ortopedico Rizzoli (Bologna, Italy). P-glycoprotein expression was evaluated using immunohistochemistry with the monoclonal antibodies JSB-1 (Monosan Sanbio, Uden, The Netherlands), MRK16 (MyBioSource Aurogene Srl, Roma, Italy), and C494 (Invitrogen, Invitrogen Ltd., Paisley, UK), each targeting non-overlapping epitopes of the protein [38,39]. A semi-quantitative scoring system was used to identify patients with increased P-glycoprotein expression levels at clinical onset, classifying them as P-glycoprotein-positive.

Standard adjuvant MAP chemotherapy was administered to patients with negative P-glycoprotein. Mifamurtide was administered to patients with positive P-glycoprotein and tumor necrosis ≥ 90%, while high-dose ifosfamide plus mifamurtide was administered to patients with positive P-glycoprotein and necrosis < 90%.

OS was calculated as the time interval between diagnosis and death from any cause or to the date of the last clinical evaluation. RFS was calculated as the time interval between the start of MAP chemotherapy and the first occurrence of either local or distant recurrence. The pathological response following neoadjuvant MAP chemotherapy was categorized as a good response when tumor necrosis ≥ 90%, and as a poor response when tumor necrosis < 90%.

### 4.2. Selection and Genotyping of Polymorphisms

Nine variants from the *VEGFA* (VEGF; rs1570360, rs2010963 and rs699947), *FLT1* (VEGFR1; rs7993418, rs9513070 and rs9582036) and *KDR* (VEGFR2; rs1551641 and rs1870377 and rs2071559) genes were prioritized for analysis (Table 2). These genetic variants have been associated with disease susceptibility, prognosis, or treatment outcomes in various types of cancer including osteosarcoma in previous studies.

Peripheral blood samples were obtained from all participants. DNA isolation was performed through automated extraction (Autopure LS system, Qiagen, Hilden, Germany). Single-nucleotide polymorphism (SNP) analysis was performed on a 7900 HT Real-Time PCR System (Applied Biosystems, Foster City, CA, USA) using TaqMan^®^ SNP assays, as specified by the manufacturer. Allelic discrimination was successful for all samples and SNPs. Quality control showed allele frequencies consistent with European reference populations [46] and confirmed Hardy–Weinberg equilibrium for all the SNPs.

### 4.3. Statistical Analysis

Data on death and on disease progression from four and six patients, respectively, was unavailable and were excluded from the respective analyses. Survival curves and median values were estimated using the Kaplan–Meier method, and survival differences between groups were estimated using the log-rank test. Codominant, dominant, and recessive genetic models were applied as appropriate. Multivariate Cox proportional hazards regression models were estimated for each significant genetic variant, including surgical margins and pathological response as covariates, and the results are reported as hazard ratios (HRs) and 95% confidence intervals (CIs). The association between genetic markers (*FLT1* rs7993418 and rs9582036 variants, and *KDR* rs1551641 variant) and pathological response was analyzed using Chi-square tests. Linkage disequilibrium strength between *FLT1* rs7993418 and rs9582036 variants was assessed using r^2^ and D′ statistics. The study sample had 74–88% statistical power to identify allelic effect sizes of f = 0.25–0.30, respectively (α = 0.05, calculated with G*Power version 3.1.9.4). The Bonferroni method was applied to correct for multiple comparisons (*p* < 0.002). SPSS Statistics (IBM, version 29.0) and the PLINK statistical package (v1.07.2) were used for statistical analyses.

## Figures and Tables

**Table 1:** Significant associations between genetic variants and survival in high-grade osteosarcoma patients.

**Recurrence-Free Survival**					
**SNP**	**n**	**5-Year Probability (%) (95% CI)**	*p*-value * (log-rank)	**Unadjusted HR (95% CI)**	**Adjusted HR (95% CI)**
*FLT1* rs7993418			**0.03**		
TT	36	89 (79–99)		1	
TC	24	48 (21–75)		4.17 (1.31–13.32)	
CC	3	50 (NA-NA)		4.65 (0.51–42.18)	
TC-CC a	27	49 (24–74)	**0.01**	4.21 (1.34–13.25)	3.71 (1.15–12.01)
*FLT1* rs9582036			0.07		
AA	25	92 (82–NA)		1	
AC	32	58 (34–82)		4.71 (1.04–21.26)	
CC	6	57 (12–NA)		5.52 (0.77–39.75)	
AC-CC a	38	58 (36–80)	**0.02**	4.81 (1.08–21.36)	4.84 (1.08–21.73)
*KDR* rs1551641			**0.01**		
CC	29	79 (63–95)		1	
CT	28	74 (50–98)		0.83 (0.25–2.74)	
TT	6	33 (NA–70)		4.78 (1.31–17.41)	
CC-CT b	57	76 (60–92)	**0.002**	0.19 (0.06–0.62)	7.34 (2.07–26.03)
**Overall Survival**					
**SNP**	**n**	**5-Year Probability (%) (95% CI)**	*p*-Value * (log-rank)	**Unadjusted HR (95% CI)**	**Adjusted HR (95% CI)**
*FLT1* rs7993418			**0.01**		
TT	38	79 (61–97)		1	
TC	24	68 (44–92)		1.71 (0.55–5.34)	1.33 (0.4–4.46)
CC	3	33 (NA–86)		8.51 (1.65–43.85)	10.38 (1.86–57.88)
*FLT1* rs9582036			**0.03**		
AA	27	84 (70–98)		1	
AC	32	68 (46–90)		1.41 (0.41–4.85)	2 (0.34–4.27)
CC	6	46 (4–87)		6.12 (1.30–28.74)	7.22 (1.43–36.42)

a Dominant model; b Recessive model. SNP, Single-nucleotide polymorphism; HR, hazard ratio; CI, confidence interval. * Uncorrected *p*-values. Statistically significant *p*-values are marked in bold.

**Table 2:** Details of the selected genetic variants.

rsID	Gene	Consequence	Alleles	MAF (Allele)	References PGx
rs1570360	*VEGFA*	2KB Upstream	A/G	0.32 A	[40,41]
rs2010963	*VEGFA*	5′-UTR	C/G	0.33 C	[18,32,33,42]
rs699947	*VEGFA*	2KB Upstream	A/C	0.48 C	[18,33,41]
rs7993418	*FLT1*	Stop Gained	T/C	0.20 C	[23]
rs9513070	*FLT1*	Intron	G/A	0.42 G	[31]
rs9582036	*FLT1*	Intron	C/A	0.27 C	[22,23,27,28,43]
rs1551641	*KDR*	Intergenic	C/T	0.31 T	[44]
rs1870377	*KDR*	Missense	T/A	0.24 A	[27,33,45]
rs2071559	*KDR*	2KB Upstream	A/G	0.49 G	[32]

rsID, Reference Single-nucleotide polymorphism Identifier; MAF, minor Allele Frequency; PGx, Pharmacogenetics; *VEGFA*, Vascular Endothelial Growth Factor; *FLT1*, Fms-Related Receptor Tyrosine Kinase 1; *KDR*, Kinase Insert Domain Receptor.

## Data Availability

In accordance with ethical committee regulations, the data from this study are not publicly available, but they can be provided on request.

## Supplementary Materials

The following supporting information can be downloaded at: https://www.mdpi.com/article/10.3390/ph18121855/s1, Table S1: Osteosarcoma patient clinical and pathological data; Table S2: Univariate associations between genetic variants and recurrence-free survival in high-grade osteosarcoma patients; and Table S3: Univariate associations between genetic variants and overall survival in high-grade osteosarcoma patients.

## Abbreviations

VEGF	Vascular Endothelial Growth Factor
VEGFR1	Vascular Endothelial Growth Factor Receptor 1
VEGFR2	Vascular Endothelial Growth Factor Receptor 2
*FLT1*	Fms Related Receptor Tyrosine Kinase 1
*KDR*	Kinase Insert Domain Receptor
GEIS	Spanish Research Sarcoma Group
