# Supplementary material for: VEGF Signaling Pathway Germline Polymorphisms as Prognostic Pharmacogenetic Biomarkers in Localized High-Grade Osteosarcoma Patients from the GEIS-33 Protocol
Source: Pharmaceuticals (Basel). 2025 Dec 5;18(12):1855. doi: 10.3390/ph18121855 (PMC12735774; doi:10.3390/ph18121855)
Supplement: Supplementary file 1 [file pharmaceuticals-18-01855-s001.zip › pharmaceuticals-4009970-supplementary.pdf]

SUPPLEMENTARY MATERIAL

VEGF Signaling Pathway Germline Polymorphisms as Prognostic Pharmacogenetic Biomarkers in Localized High-Grade Osteosarcoma Patients from the GEIS-33 Protocol

Juliana Salazar [1,*], María J. Arranz [2], Javier Martin-Broto [3], Massimo Serra [4], Emanuela Palmerini [4,5], Jeronimo Garcia [6], Antonio Juan-Ribelles [7], Maitane Andion [8], Javier Martinez Trufero [9], Guiomar Gutiérrez [10], Aizpea Echebarria-Barona [11], Maria A. Vaz Salgado [12], Alexandra Regueiro [13], Rosa Alvarez [14], Claudia Valverde [15], Pablo Luna [16], Pilar Blay [17] and Ana Sebio [1,18,*]

Table S1. Osteosarcoma patient clinical and pathological data.

**Characteristic**	**N (%)**
**Age at diagnosis** (years), median (range)	14 (4-32)
**Sex**	
Female	32 (46.4)
Male	37 (53.6)
**Primary tumour site**	
Femur	45 (65.2)
Tibia/fibula	12 (17.4)
Humerus/radius	8 (11.6)
Other	4 (5.8)
**Surgical margins**	
Wide/ radical	51 (74)
Marginal	15 (21.7)
Unknown	3 (4.3)
**Pathological response**	
≥90	26 (37.7)
<90	40 (58)
not available	3 (4.3)
**Death**	14 (21.2)
**Progression**	16 (23.9)

Progression was defined as the development of lung metastases. Pathological response to neoadjuvant MAP chemotherapy was classified into good response (tumor necrosis ≥ 90%) and poor response (tumor necrosis < 90%).

Table S2. Univariate associations between genetic variants and recurrence-free survival in high-grade osteosarcoma patients.

Recurrence-free survival				
**SNP**	**n**	**5-Year Probability (%) (95% CI)**	**Unadjusted HR (95% CI)**	***p*-value* (log-rank)**
VEGFA rs1570360				0.22
GG	27	77 (61 - 93)	1	
GA	31	80 (66 - 94)	0.88 (0.28 - 2.72)	
AA	5	0 (0 - 0)	2.71 (0.68 - 10.84)	
VEGFA rs2010963				0.97
GG	24	65 (38 - 92)	1	
GC	34	76 (62 - 90)	0.92 (0.32 - 2.66)	
CC	5	78 (39 - NA)	0.77 (0.09 - 6.39)	
VEGFA rs699947				0.38
CC	14	85 (65 - NA)	1	
CA	39	76 (62 - 90)	1.70 (0.37 - 7.87)	
AA	10	34 (NA - 83)	3.08 (0.56 - 16.83)	
FLT1 rs7993418				**0.03**
TT	36	89 (79 - 99)	1	
TC	24	48 (21 - 75)	4.17 (1.31 - 13.32)	
CC	3	50 (NA- NA)	4.65 (0.51 - 42.18)	
TC - CC [a]	27	49 (24 - 74)	4.21 (1.34 - 13.25)	**0.01**
FLT1 rs9513070				
AA	17	87 (71 - NA)	1	0.2
AG	34	70 (48 - 92)	2.16 (0.46 - 10.17)	
GG	12	57 (28 - 86)	4.02 (0.78 - 20.78)	
FLT1 rs9582036				0.07
AA	25	92 (82 - NA)	1	
AC	32	58 (34 - 82)	4.71 (1.04 - 21.26)	
CC	6	57 (12 - NA)	5.52 (0.77 - 39.75)	
AC -CC [a]	38	58 (36 - 80)	4.81 (1.08 - 21.36)	**0.02**
KDR rs1551641				**0.01**
CC	29	79 (63 - 95)	1	
CT	28	74 (50 - 98)	0.83 (0.25 - 2.74)	
TT	6	33 (NA - 70)	4.78 (1.31 - 17.41)	
CC -CT [b]	57	76 (60 - 92)	0.19 (0.06 - 0.62)	**0.002**
KDR rs1870377				0.52
TT	47	73 (59 - 87)	1	
TA-AA	16	68 (33 - NA)	0.66 (0.19 - 2.36)	
KDR rs2071559				0.22
GG	13	58 (31 - 85)	1	
GA	37	72 (52 - 92)	0.44 (0.14 - 1.38)	
AA	13	85 (65 - NA)	0.29 (0.06 - 1.56)	

[a] Dominant model; [b] Recessive model. *Uncorrected *p*-values. SNP, Single-nucleotide polymorphism; HR, hazard ratio; CI, confidence interval. Statistically significant *p*-values are marked in bold.

Table S3. Univariate associations between genetic variants and overall survival in high-grade osteosarcoma patients.

Overall survival				
**SNP**	**n**	**5-Year Probability (%) (95% CI)**	**Unadjusted HR (95% CI)**	***p*-value* (log-rank)**
VEGFA rs1570360				0.61
GG	27	72 (48 - 96)	1	
GA	33	78 (64 - 92)	1.29 (0.41 - 4.06)	
AA	5	39 (NA - 96)	2.27 (0.43 - 11.89)	
VEGFA rs2010963				0.6
GG	26	70 (48 - 92)	1	
GC	34	71 (51 - 91)	0.99 (0.34 - 2.87)	
CC	5	100 (100 - 100)	0.99 (NA - NA)	
VEGFA rs699947				0.31
CC	14	91 (75 - NA)	1	
CA	40	69 (51 - 87)	4.21 (0.54 - 32.69)	
AA	11	64 (19 - NA)	2.84 (0.26 - 31.60)	
FLT1 rs7993418				**0.01**
TT	38	79 (61 - 97)	1	
TC	24	68 (44 - 92)	1.71 (0.55 - 5.34)	
CC	3	33 (NA - 86)	8.51 (1.65 - 43.85)	
FLT1 rs9513070				0.3
AA	18	83 (65 - NA)	1	
AG	34	77 (59 - 95)	1.07 (0.27 - 4.28)	
GG	13	45 (6 - 84)	2.42 (0.58 - 10.16)	
FLT1 rs9582036				**0.03**
AA	27	84 (70 - 98)	1	
AC	32	68 (46 - 90)	1.41 (0.41 - 4.85)	
CC	6	46 (4 - 87)	6.12 (1.30 - 28.74)	
KDR rs1551641				0.13
CC	30	70 (48 - 92)	1	
CT	28	79 (57 - NA)	0.60 (0.18 - 2.06)	
TT	7	57 (20 - 94)	2.61 (0.65 - 10.41)	
KDR rs1870377				0.49
TT	47	69 (51 - 87)	1	
TA-AA	18	83 (65 - NA)	0.64 (0.18 - 2.30)	
KDR rs2071559				
GG	14	71 (47 - 95)	1	0.42
GA	38	74 (56 - 92)	0.52 (0.15 - 1.80)	
AA	13	73 (38 - NA)	0.35 (0.06 - 2.00)	

*Uncorrected *p*-values. SNP, Single-nucleotide polymorphism; HR, hazard ratio. Statistically significant *p*-values are marked in bold.
